# Thermal Effects on Mechanical Strength of Additive Manufactured CFRP Composites at Stable and Cyclic Temperature

**DOI:** 10.3390/polym14214680

**Published:** 2022-11-02

**Authors:** Isyna Izzal Muna, Magdalena Mieloszyk, Ruta Rimasauskiene, Nabeel Maqsood, Marius Rimasauskas

**Affiliations:** 1 Institute of Fluid Flow Machinery, Polish Academy of Sciences, Fiszera 14, 80-231 Gdansk, Poland; 2 Department of Production Engineering, Faculty of Mechanical Engineering and Design, Kaunas University of Technology, Studentu 56, 51424 Kaunas, Lithuania

**Keywords:** thermal cycle, thermally stable, 3D-printing technology, fused deposition modeling, CFRP composites, mechanical degradation, morphological structure

## Abstract

Additive manufacturing (AM) techniques can be applied to produce carbon-fiber-reinforced polymer (CFRP) elements. Such elements can be exposed to different environmental factors, e.g., temperature, moisture, and UV radiation, related to their operational conditions. From a variety of environmental factors, the temperature is one of the most typical. Temperature strongly influences matrix material joining together CFRP components, resulting in material strength reduction. Therefore, it is important to understand processes in the composite material caused by temperature. This experimental work investigated the thermal effects on the performances of AM CFRP composites. Specimens with unidirectional (UD) alignments of the fiber reinforcement were printed using the fused deposition modeling (FDM) technique. The printed specimens were subjected to two different thermal conditions: stable continuous at 65 °C and cyclic temperature between 50 and 70 °C. Tensile testing was performed to study the mechanical strength and Young’s modulus of AM UD-CFRPs. In order to investigate the morphological structure on the surface of AM specimens, an optical microscope, scanning electron microscope (SEM), and digital microscope were utilized. Untreated (intact) samples attained the highest average tensile strength value of 226.14 MPa and Young’s modulus of 28.65 GPa. The ultimate tensile strength of the sample group subjected to stable heat treatment decreased to 217.99 MPa, while the thermal cycling group reduced to 204.41 MPa. The Young’s modulus of the sample group subjected to stable thermal exposure was decreased to 25.39 GPa, while for the thermal cycling group, it was reduced to 20.75 GPa. The visual investigations revealed that the intact or untreated specimen group exhibited lateral damage in top failure mode (LAT), the thermally stable group underwent edge delamination in the middle (DGM) as the nominated failure mode, and the explosive breakage at gauge in the middle (XGM) failure mode occurred in the sample from the thermal cycling group. Based on morphological observations at the microscale, the delamination, fiber pull-out, and matrix cracking were the dominant damages in the 3D-printed tensile-tested specimens. The molecular chains of the polymer changed their structure into an amorphous one, and only local motions of stretching occurred when the specimens were exposed to stable heating (prolonged). In the case of thermal cycling, the strain gradients were accumulated in the matrix material, and the local stresses increased as a result of the reheating and re-cooling exposure of the polymeric composites; the molecular motion of the long-range polymer structure was reactivated several times. Micro-cracking occurred as a result of internal stresses, which led to material failure and a reduction of the mechanical properties.

## 1. Introduction

The technological advancement in additive manufacturing (AM) over the last decade has been a major driver for the development of composite materials. It is widely known that AM technologies, specifically the fused deposition modeling (FDM) method, have numerous benefits owing to the low price, flexibility, and simplicity with small amounts of waste material, which indeed open up endless possibilities in their utilization in engineering and industrial fields [1,2,3]. Carbon-fiber-reinforced polymer (CFRP) has potential applications in the industrial field such as automotive bumper brackets [4] and the development of more durable wind turbines [5]. The printed composite materials must have a long service life in real applications. However, these materials are more likely exposed to harsh environmental conditions specifically related to temperature. Depending on its different magnitude and period time, the exposure to temperature of 3D-printed composites could affect the mechanical behavior of the materials differently. Researchers usually use different thermal cycling profiles to study the behavior of aerospace vehicles such as satellite components, which depend on their case study or thermal cycling test apparatus [6].

There have been a large number of papers that experimentally studied the effect of thermal treatment of the mechanical behavior of polymeric composites manufactured with the FDM method at a continuous temperature [7,8,9,10,11,12]. Several results reported that the heat treatment leads to the better mechanical properties of 3D-polymer-based composites [10,11,12]. Handweker et al. investigated the influence of a heat treatment (annealing) process on the mechanical performance of continuous- and chopped-fiber-reinforced polyamide 6 in the build-up direction. It was found that Young’s modulus increased by a factor of three, while the ultimate tensile strength (UTS) increased by 50% for the chopped-carbon-fiber-reinforced material and 186% for the continuous-glass-fiber-reinforced material [11]. In Wang et al. [12], heat treatment was performed on three printed continuous-carbon-fiber-reinforced polymer (CCFRP) composite specimens with different layer distributions and heating conditions. The dimensional change of CCFRCs during heat treatment was shown to be much more closely related to the microstructure change than the material crystallization. Furthermore, heat treatment could improve the mechanical properties of CCFRCs by decreasing the porosity while also strengthening the interface. Heat treatment, specifically, delayed the initiation of cracks in CCFRCs during bending tests by changing the failure mode of the matrix layers and improving the bonding between fiber and bundles/matrix. Another experimental work by Nassar et al. revealed that heat treatment was able to enhance the bonding of filament layers and reduce the porosity content of CFRP composite printed using the FDM method, which resulted in an improvement of its tensile modulus. However, there was a limited improvement in the tensile strength and modulus of elasticity values for the samples treated at low heat treatment temperature [10]. Despite the benefits of using FDM to generate durable materials with high mechanical properties, this method is also known to have some limitations, such as nozzle clogging [13,14] and the appearance of satellite droplets [15].

However, some other experiments on CFRP composites showed a decreased mechanical behavior after thermal treatment [7,16]. Zhang et al. investigated the effect of high temperature on pure epoxy resin and 3D carbon/epoxy braided composites [7]. It was revealed that a constant decline in compressive properties occurred as a result of the combination of matrix degradation and fiber/resin interface cracking. At temperatures lower than the glass transition temperature (Tg) of the pure epoxy resin, the matrix aging was the main aging process, although fiber/matrix interface debonding can be detected at temperatures greater than the Tg, such as 180 ${}^{\circ }$C. After thermal aging, their micro-morphologies and compressive behaviors were reported. Jia et al. experimentally investigated the influence of heat treatment performed at a stable temperature on CFRP composites generated conventionally, ranging from −100 ${}^{\circ }$C to 100 ${}^{\circ }$C [16]. The results showed that, under static and dynamic three-point bending tests, CFRP composites had improved flexural strength, maximum deflection, and energy absorption at lower temperatures (−60 ${}^{\circ }$C, −100 ${}^{\circ }$C), but performed poorly at higher temperatures (100 ${}^{\circ }$C). At various temperatures, experimental photos from post-mortem photographs, scanning electron microscopy, and high-speed films were used to investigate various failure behaviors such as micro-buckling, kinking, and fiber breakage. In more recent experimental research by [6], 3D-printed CF/PEEK was exposed to thermal cycling and then evaluated using tensile and arc heating tests. It was reported that the thermal cycle resulted in decreased tensile strength and the length of the samples increased after the heating test.

Until recently, the number of experiments of thermal treatment conducted in another thermal mode, e.g. cyclic temperature, remains very limited since most heat treatment experiments on 3D-printed composites were mostly performed at a stable continuous temperature. However, some papers investigated the effect of thermal cycling at low temperatures on composites produced conventionally. Ghasemi et al. studied the residual stress during thermal cycling on a graphite/epoxy composite [17]. The cycle was performed one time and used as a change in temperature from 70 ${}^{\circ }$C to 100 ${}^{\circ }$C and back to 70 ${}^{\circ }$C. This profile has a heating/cooling (transition) rate of 3–5 ${}^{\circ }$C per minute, while the duration of the dwelling time at the temperature extremes was about 15 min. It was reported that the mechanical properties of this composite were degraded when the material was exposed to thermal cycling conditions. Up to a certain period of exposure, the composite may retain its strength and stiffness above its allowable limits. However, as time passes, the strength and stiffness may become very low.

Another thermal cycling treatment of a carbon fiber/epoxy composite generated conventionally was carried out by Gonzalez et al. [18]. On CFRP samples, 500 thermal cycles at 15 ${}^{\circ }$C/min and a 5 min dwell time were applied during the experimental investigation. Two different atmospheres were used in the thermal cycling test. The first represented an inert atmosphere (nitrogen gas) for isolating any oxidative agents’ effects. For the second atmosphere, dry air was used to simulate material oxidation effects. At high temperatures, matrix oxidation was expected, but matrix cracking occurred due to a mismatch in the thermal expansion coefficient. The combination of both effects resulted in CFRP aging acceleration. Lafarie et al. studied the characterization of the damage processes of carbon/epoxy laminates under isothermal aging and thermal cycling conditions in a vacuum and oxidative atmosphere [19]. The thermal cycling experiments were 500 thermal cycles with maximum and minimum temperatures of 180 and −50 ${}^{\circ }$C, respectively, and constant cooling and heating rates of 4 ${}^{\circ }$C/min. It was found that, during a thermal cycling test in an oxidative atmosphere such as air, there was a coupling effect between matrix oxidation, which occurs at the highest temperatures of the cycle, and matrix cracking caused by thermo-mechanical ply stresses caused by prohibited differential expansions of the plies. Previously, heating treatment of unidirectional (UD) continuous CFRP composite at an elevated temperature was experimentally and numerically investigated by Muna et al. [20]. The coupled thermal–displacement analysis was used during the numerical calculations. The strain in the sample due to its exposure to elevated temperature was measured using fiber Bragg grating (FBG) sensors.

Thermal degradation of additive manufactured polymer composite has not been explored by researchers comprehensively, yet it is an important phenomenon that will be useful in the structural health prediction of lightweight materials. Up to the present, the majority of research studies on the thermal degradation of 3D-printed composites have only been investigated on specimens at a stable temperature for a certain duration, while for the thermal cycling treatment, most of the research work has been performed on the specimens produced not with 3D-printing technology, but rather, conventionally. Furthermore, few explorations are available on the work performed on 3D-printed polymeric composites under both thermal conditions at continuous and cyclic temperatures and the effect on the mechanical degradation. This paper aims to investigate the effect of thermal exposure on the mechanical and morphological behavior of 3D continuous-carbon-fiber-reinforced polymers at stable continuous and cyclic temperatures.

## 2. Experimental Work

### 2.1. Additive Manufacturing of CFRP Composite Samples

Additive manufacturing (AM) is a well-known processing technique for adding materials to manufacture parts based on a 3D computer model in a layer-by-layer manner, which can also be referred to as 3D printing or rapid prototyping (RP). In this experimental work, the FDM method was chosen due to its simplicity and ability to print complex geometries with low cost and flexibility to use different materials. Unidirectional (UD) continuous CFRP composites were printed as experimental specimens. The benefit of employing continuous carbon fibers (CCFs) as a reinforcement agent in polymer-based composites is that their weight remains light with the increased strength. Thermoplastic polylactic acid (PLA) was chosen as the matrix agent.

The CFRP composite specimens were additively manufactured with the fused deposition modeling (FDM) method at Kaunas University of Technology. A modified 3D printer MeCreator2 with dual inputs at the extrusion printing head, which was designed and developed by Rimasauskas et al., was employed [14]. The pre-impregnation process was applied for the CCF reinforcement for better printing quality and adhesion. For the impregnation process, PLA pellets were dissolved in dichloromethane solution from Eurochemicals with the ratio concentration of 90 g/10 g using the magnetic LBX H01 mini-stirrer at 600 rpm. Later, the standard non-impregnated CCF was passed through this resin and simultaneously dried with a hot air gun temperature of 220 ${}^{\circ }$C. After the impregnation process, the CCF was further printed with the PLA matrix filament to form a composite specimen. The continuous carbon fiber used was T300D-3000, consisting of 3000 fibers in an untwisted tow (Toray, France). The materials’ properties are shown in Table 1. The information of all materials concerning the mechanical properties was provided by the material suppliers. The 3 K CCF was used as the reinforcement material and was kept the same throughout the experimental work. An extrusion multiplier was used to control the content, but it was also kept the same at 0.5 for all the manufactured composite specimens. The prepared materials were then ready to be used for additive manufacturing process with the FDM method. The 3D model of the specimens was created in a CAD package, which was then exported as a stereo lithography (STL) file and subsequently loaded into 3D printer slicing software (Simplify3D, Cincinnati, OH, USA) to generate the G-code, which the 3D printer used to print out each test specimen. The CAD files of the specimens were used to extrude and deposit molten thermoplastic, which was built up in layers from a horizontal base. Two types of FDM printers were used during the experiments, a modified one with two inputs at the extruder and another 3D printer with one input from Prusa company (for printing the gripping tabs). The printing parameters are shown in Table 2.

The extrusion printing was updated in order to include two inputs (one for the polymer filament and the other for the carbon fiber tow) and one output that allowed the polymer to be fused with the CF for printing. As was discussed previously in the experimental research by [14], the working principle of PLA and CF mixing consisted of several steps. Firstly, PLA filament was fed through a PTFE tube by using a standard filament feeding system. When the filament reached the printing head, which was heated up to 200 ${}^{\circ }$C, the polymer liquefied. Subsequently, the impregnated carbon fiber tow was inserted through the PTFE tube to the printing head, directly to the printing nozzle. The molten polymer came into contact with the carbon fiber in the mixing zone, and secondary carbon fiber impregnation was performed. The molten polymer was constantly pushed together with the carbon fiber through the nozzle. It should be emphasized that the carbon fiber was fed solely by the molten polymer and did not require any additional feeding equipment. Later, the carbon fiber coated with polymer was extruded through a printing nozzle onto borosilicate glass mounted on an aluminum plate.

Generally, the manufacturing process of CFRP composites with the FDM method requires a long period of time (e.g., in this experimental case, about 1 h for printing 1 specimen), and the 3D printer is not designed to handle errors. Printing mistakes are frequently expected, and continuing the printing process when an error appears may endanger the 3D printer, and the final part of the printed sample typically cannot be used. One common printing error is nozzle clogging [13,14]. In order to minimize this detrimental issue, numerous efforts have been made by researchers to overcome nozzle clogging during the printing process [13,21,22]. Gutierrez et al. proposed a method to decrease the clogging deposition rate of alumina inclusions in continuous casting nozzles through three simultaneous measures: flow modification, the use of raw materials with a low impurity content, and smoothed internal surfaces [13]. Internal flow regulation entails avoiding dead zones and generating symmetric patterns. The feasibility of these methods was tested using a mathematical model. Using this approach, the adherence of inclusions to the nozzle wall applied a boundary condition based on the thickness of the sub-laminar boundary, rather than the typical “trap” boundary condition. The generic boundary condition produces deposition rates that are unaffected by the inclusion size. The proposed boundary condition uses particle sizes to distinguish against the clogging deposition rate. Plant studies with these nozzles, together with water modeling showed that the current strategy may significantly reduce clogging occurrence. More recently, as a response to the problem of nozzle clogging, Sampedro et al. created a real-time process monitoring system capable of accurately forecasting abnormal behaviors in the FDM printing method [21]. A network of collaborative sensors was utilized to collect time series data, which were then processed by the suggested machine learning method. The multi-head encoder–decoder temporal convolutional network (MH-ED-TCN) gathers features from the data, assesses their impact on the many processes that occur throughout an operational printing cycle, and distinguishes between normal and problematic manufacturing operations. The studies conducted yielded a 97.2% accuracy in predicting the future behavior of a 3D printer. More recent experimental work has been conducted to improve the quality of the 3D-printed composite specimen using a controlled cooling effect [23]. In regard to achieving this printing quality, the effect of printing parameters (extrusion temperature, extrusion width, and extrusion multiplier) on the tensile and flexural properties were examined. The highest Young’s and flexural modulus of 31.50 GPa and 15.68 GPa, respectively, were reached when the composite structure was manufactured using printing parameters E.W. −1.3 mm and E.M. −0.5.

**Figure 1:**
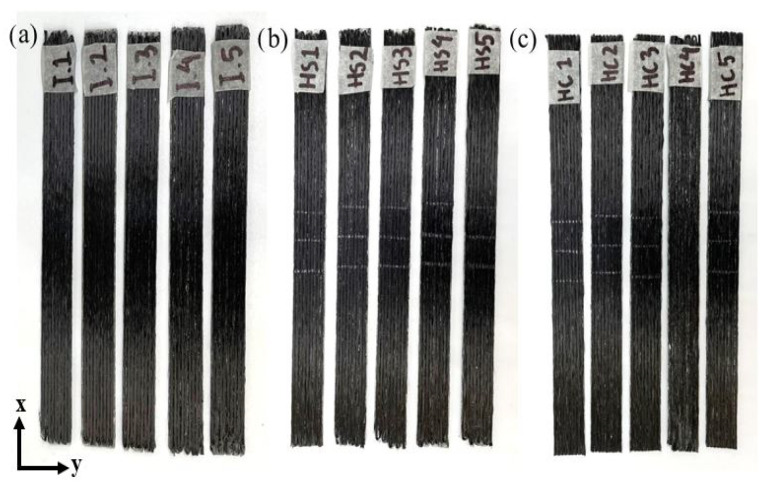
The 15 CFRP composite specimens printed with the FDM method for (**a**) intact/no treatment, (**b**) continuous temperature, and (**c**) cyclic temperature.

Next, thermoplastic PLA reinforced with CF composite was extruded through the printing nozzle, which was printed on borosilicate glass mounted on the aluminum plate bed surface at a certain temperature according to the optimized printing parameters in the literature (i.e., 90 ${}^{\circ }$C). Before starting the printing process, adhesive spray should be spread out on top of the borosilicate glass to avoid the dragging of printed filaments. Once the printing of the sample completed, a cutter was used to separate the filament spool and printed part. Subsequently, the borosilicate glass should be removed from the aluminum plate bed and to allowed cool down, then the 3D-printed sample can be removed from the glass by the aid of blades. The dimension of unidirectional (UD) CFRP composites was 150 × 13 × 2 mm with 6 layers of the same fiber alignment in all layers. The volume fraction of the composite was kept constant throughout the experimental work due to having the same printing parameters. The carbon fiber reinforcement content was measuring using the tool length path, and the estimated carbon fiber content was 18.2% (wt.). The total number of specimens printed was 15 samples in which 5 samples for each group of thermal treatment were required in accordance with the D3039 ASTM standard used for tensile testing. The specimens are depicted in Figure 1. After the manufacturing stage completed, the weight and size dimensions of the composite parts were measured using a digital scale and a caliper, respectively. The weight and dimension measurement was necessary in order to compare the changes in size and mass before and after thermal loading. Furthermore, it was important for the calculation of the mechanical properties after the mechanical testing.

### 2.2. Thermal Treatment on Printed Specimens

**Figure 2:**
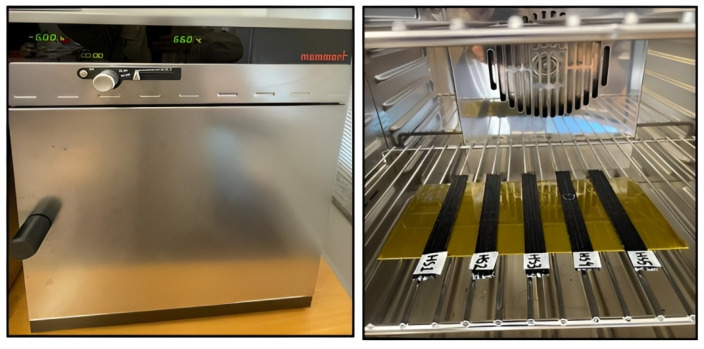
An air-circulated oven for hot temperature treatment.

**Figure 3:**
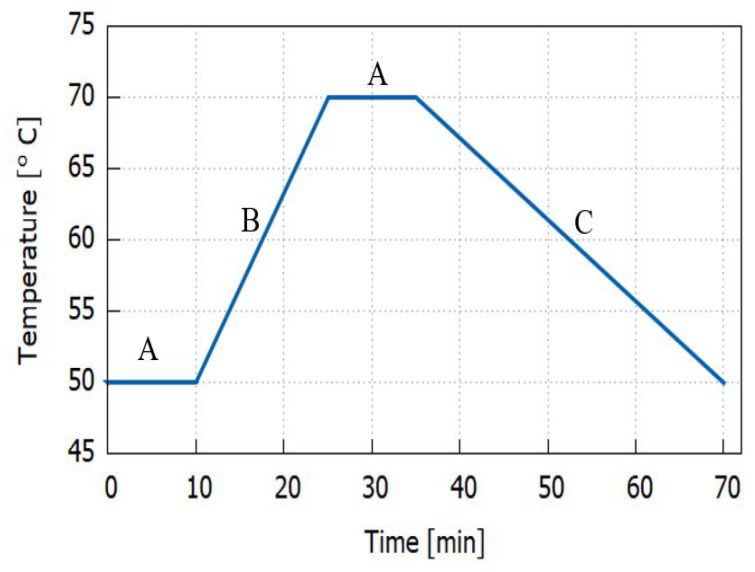
Thermal cycling profile for one cycle. A = dwell time; B = heating rate; C = cooling rate.

In an attempt to investigate the effect of temperature exposures on the printed CFRP composites via FDM, the specimens were thermally treated under two different thermal conditions: stable continuous and cyclic at low temperature around the glass transition (Tg) of the PLA polymers used in this work. An air-circulated environmental oven “Memmert” Model UFB-400 was utilized for stable and cyclic heating, which supplied a continuous renewal of oxygen in a certain percentage of ambient air, as presented in Figure 2. For the stable continuous heat treatment, the specimens were placed inside the pre-heated oven at 65 ${}^{\circ }$C and kept for 6 h, then naturally cooled at room temperature. As for the thermal cycling, the oven temperature was manually adjusted from 50 to 70 ${}^{\circ }$C according to the cyclic plan (see Figure 3) for 6 cycles. One cycle consisted of two extreme temperatures with a dwell time of 10 min, a heating rate of 1 ${}^{\circ }$C/min, and a cooling rate of 2.5 ${}^{\circ }$C/min. These temperatures were chosen according to the glass transition temperature of the PLA used in this work, which lies around 65 ${}^{\circ }$C. It was expected that the specimens would undergo cross-linking and a crystallization change of the polymer up to the state where its structural shape would particularly deform.

Dwell time means how long of an exposure period is appropriate at each temperature limit. If a prolonged dwell time is employed, the duration of the test will lengthen until the number of cycles decreases. Then, again, in some instances, a long dwell period can potentially speed up the testing process if it generates structural changes that increase the stresses during temperature fluctuations. At high temperatures, polymer materials, for instance, relax or creep—the polymer chains within the material move to alleviate the stresses imposed by the high temperature. When the temperature is decreased, these alterations may result in a large increase in the stresses imposed on buildings. However, sufficient dwell time is necessary for these modifications to take place.

### 2.3. Static Tensile Testing

**Figure 4:**
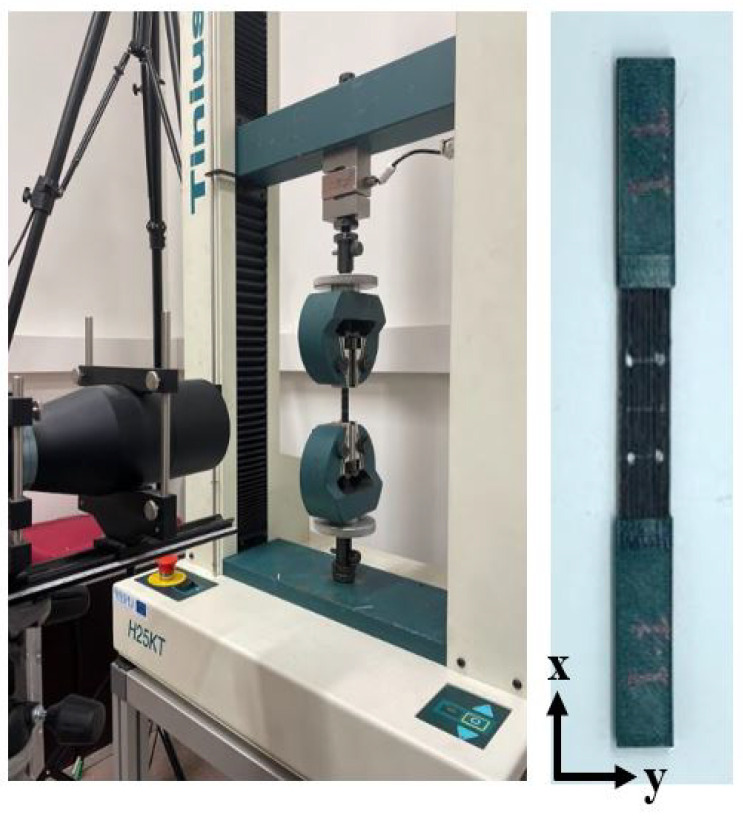
Experimental setup of tensile testing.

The tensile testing was performed after thermal treatment to examine the mechanical strength and Young’s modulus of the treated specimens, as well as the untreated samples (intact). A Tilnius Olsen H25KT (capacity 25 kN) universal testing machine was utilized to perform tensile testing with the standard head displacement speed of 2 mm/min and at room temperature. The tensile testing setup is shown in Figure 4. The ASTM standard D3039 was used for the in-plane tensile testing, which requires each tested specimen to have four tabs in gripping position at the top and bottom. Prior to the tensile testing, PLA tabs having dimensions 50 × 12.5 × 2 mm were printed separately, and four points were marked 15 mm from the center of the specimens to measure the elastic strain. The printed tabs then were adhered to the specimens with a universal structural bonder (adhesive glue) by applying pressure via clamps to hold and secure the parts tightly. In this study, the ASTM D3039 standard was used to perform the tensile testing of the specimens, in which five samples were required for each group of thermal treatment to determine the average material properties.

### 2.4. Morphological Investigation

**Figure 5:**
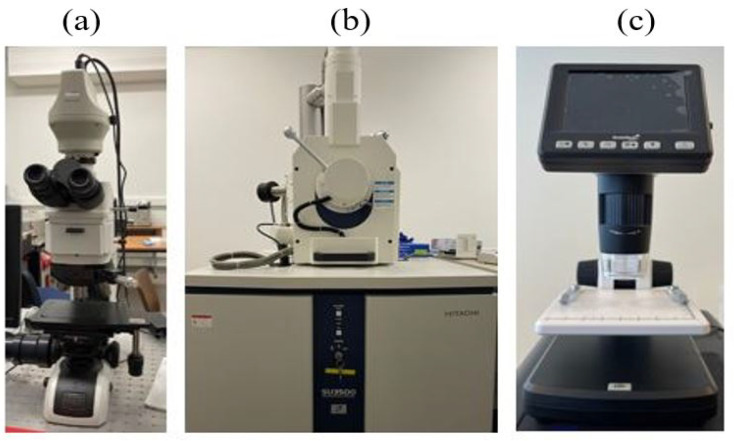
(**a**) Optical microscope; (**b**) scanning electron microscope; (**c**) digital microscope.

The micro-morphological analyses on the surface were performed before and after performing the cyclic and stable thermal loadings by utilizing an optical microscope device (Nikon Eclipse LV100ND) equipped with a high-definition color camera (Nikon DS-Ri2). The imaging software (NIS Elements 4.5.1.00, Nikon Europe B.V., Amstelveen, The Netherlands) was used to prepare and process the data at 5× magnification. The maximum sample size observed with the optical microscope was 150 × 150 mm. A scanning electron microscope device (FE-SEM SU5000, Hitachi Co., Tokyo, Japan) was employed to investigate the micro-structure damage of different specimen groups after tensile testing. The maximum specimen size observed with the SEM was 200 mm in diameter and 80 mm in height. A digital microscope (Levenhuk, DTX 500 LCD, Warsaw, Poland) was utilized to capture the macroscopic images of the tensile-fractured samples. The maximum specimen size observed with the digital microscope was about 150 × 100 mm. The microscopes are presented in Figure 5.

## 3. Results

### 3.1. Dimensional Change

The samples were measured before and after the heat treatment in order to determine the amount of geometrical changes, as shown in Table 3. Any thermal treatment is expected to have an effect on the dimensions and shape of a plastic part. For the test samples, the length, as well as the height and width were measured using a digital vernier caliper dimensional measurement tool. Table 1 shows the change of the specimens’ length, width, and thickness before heat treatment (after 3D printing) and after heat treatment. The difference between the nominal design from the CAD software and the printed dimension in practice is referred as variations of the relative values of the length, width, and thickness. These values were then acquired and averaged. In comparison to the nominal model, the dimensions of printed CFRP samples in the xy-plane (length and width) were undersized around (0.62 ± 0.93)% and (0.07 ± 0.27)%, respectively. However, in comparison with the 3D model, the dimension in the z direction (thickness) was oversized, and a mean error of 13.44 ± 0.05% was obtained. Overall, these findings are consistent with previous research on the dimensional correctness of 3D-printed composite parts [8,24]. The noticeable difference in the dimension of the specimens can be hypothetically attributed to the difficulty of depositing impregnated continuous carbon fiber filament. During the printing process, the clogging of the printer nozzle could possibly occur due to the improper proportion of polymer matrix filament mounted into the impregnated CCF material. In addition to that, the issue with nozzle clogging might lead to the breakage of the CCF filament throughout the deposition process. The material deposition issue was encountered by the authors in this experimental work. In accordance with previous experimental research, a similar problem of nozzle clogging occurred when the amount of carbon fiber filament was higher than the polymer matrix, which caused a greater tension at the nozzle tip and resulted in damaged carbon fiber filament [14]. It is worth noting that carbon fiber filament was printed according to the pre-defined path where the carbon content was calculated by evaluating the path traveled by the printing head and carbon weight per unit length. Therefore, in an attempt to tackle this issue during the materials’ deposition, the cooling fan shall be adjusted to prevent either a lack or excessive polymer extruded onto the printing bed.

### 3.2. Static Tensile Results

**Figure 6:**
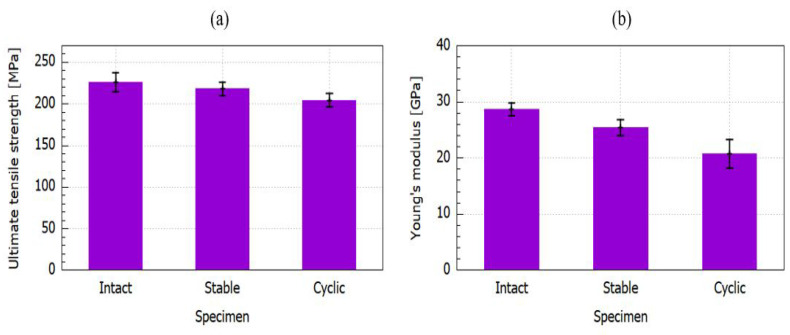
Experimental results of the tensile properties of 3D-printed samples. (**a**) Tensile strength; (**b**) Young’s modulus.

The tensile properties of the 3D-printed specimens of each thermal group (untreated, continuous, and cyclic) are shown in Figure 6. From the bar graph plot, it can be seen that the untreated (intact) samples attained the highest average tensile strength value of 226.14 MPa and Young’s modulus of 28.65 GPa. The ultimate tensile strength of the sample group subjected to stable heat treatment decreased to 217.99 MPa, while for the thermal cycling group, it reduced to 204.41 MPa. The Young’s modulus of the sample group subjected to stable thermal exposure decreased to be 25.39 GPa, while for the thermal cycling group, it reduced to 20.75 GPa. The mean values in the bar plots were used to depict the trends of each printed group specimen and the range of their tensile characteristics effects. The degradation in the mechanical strength and elastic modulus after thermally stable and cyclic loading was attributed to the difference in the coefficient of thermal expansion (CTE) between the matrix and fiber, which was caused by the reduced cross-linking of the polymers [25]. This CTE discrepancy resulted in thermal stress, and it can cause the fibers to pull out due to fiber–matrix debonding, which then leads to the mechanical deterioration of composite specimens [18,26]. ANOVA analysis was performed to identify statistical differences and significance between the unheated group and the stable continuous and cyclic temperature on the tensile strength with a significance level of 5%. A one-way ANOVA revealed that there was a statistically significant difference in tensile strength between the three tested groups with a *p*-value of 0.0108 and test statistic F of 6.764 (F(2, 27) = [6.764], *p* = 0.0108).

**Figure 7:**
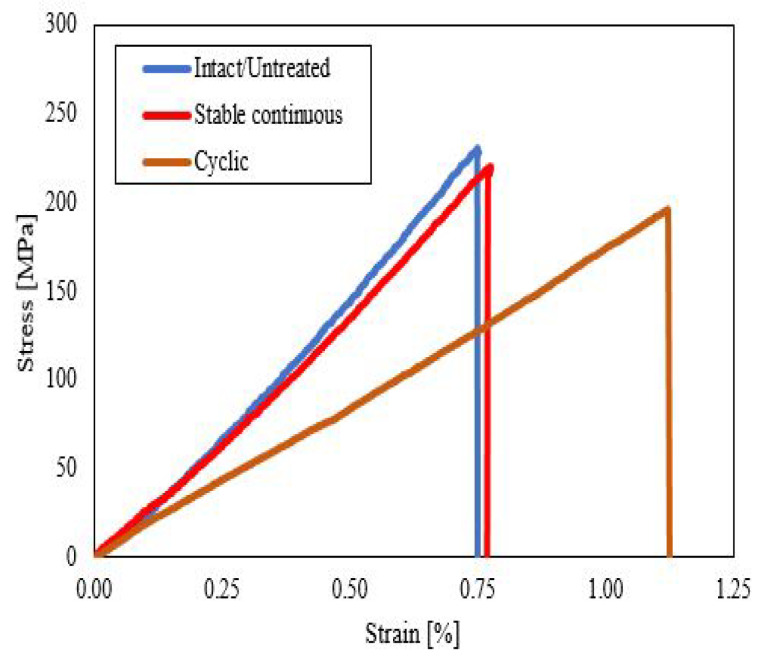
Average stress–strain curve for the tensile samples.

Figure 7 depicts the representative stress–strain curves of unidirectional CFRP composites during the static tensile tests at various thermal conditions. All of the stress–strain curves showed a linear elastic regime, followed by a stress drop. From the tensile stress–strain curves, it can be observed that the untreated/intact composite specimen reached the maximum stress level, followed by the stable continuous and cyclic composite specimens. The stable continuous 3D-fabricated composite specimen was slightly less compared to the untreated specimen with almost similar behavior in strain, while the cyclic composite specimen attained the lowest stress level among the composite group and reached beyond the strain level of an untreated and stable continuous composite specimen, which indicated its greater elasticity.

**Figure 8:**
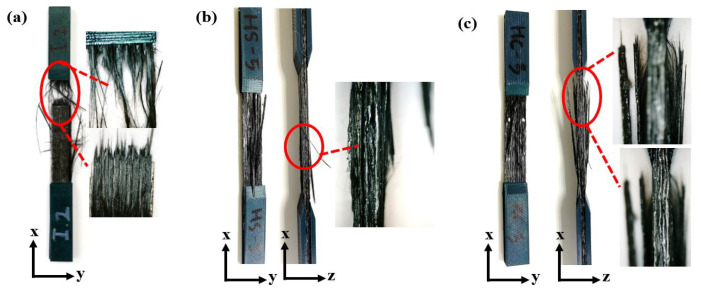
Fractured samples after performing tensile testing. (**a**) Intact; (**b**) continuous; (**c**) cyclic.

The visual investigation of the failure area of the 15 specimens was studied in accordance with ASTM standard D3039. The failure mode of each thermal group is presented in Figure 8. It was indicated that the intact or untreated specimen group exhibited the lateral-at tab-top (LAT) failure mode and well agreed with the result reported by researchers [27]. The intact sample after longitudinal tension loading exhibited lateral fiber breakage and fiber splitting completely at the top region near the tab, which is gripped by the gauge. Edge delamination in the middle (DGM) was the nominated failure mode in the stable continuous heated group’s specimen. It can be seen that there was debonding between layers in the middle due to the lower strength of the interfacial adhesion formed after the continuous thermal loading for 6 h at 65 ${}^{\circ }$C. The explosive breakage at gauge in the middle (XGM) failure mode occurred in the sample from the treated group after thermal exposure with cycling mode between 50 ${}^{\circ }$C and 70 ${}^{\circ }$C for six cycles, and this result was similar to what was studied by Ghasemi et al. [28]. In this damage mode, a large number of fibers pull out near the gauge, and this was presumed due to reduce the chain scission and cross-linking of the polymer matrix caused by thermal cycling. Moreover, it can be observed from Figure 8c that layer debonding also occurred after thermal cycling.

### 3.3. Morphological Investigation

Morphological analyses were performed before and after performing the thermal loading exposures by utilizing an optical microscope device, while a scanning electron microscope was employed to investigate the micro-structure of the different specimen groups after tensile testing. The microscopes are shown in Figure 5.

**Figure 9:**
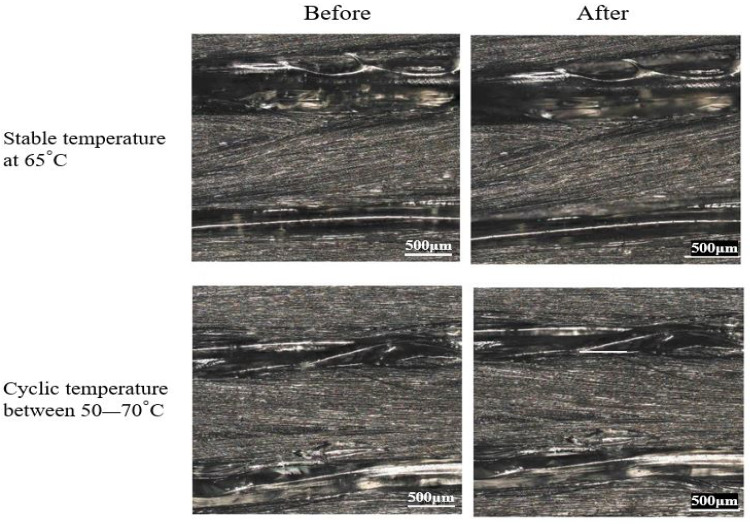
Optical micrographs of specimen before and after stable temperature at 65 ${}^{\circ }$C and cyclic temperature between 50 and 70 ${}^{\circ }$C.

In order to represent the difference on the structural surface, one specimen of each heating group before and after thermal loading was compared. It can be seen from Figure 9 that there was a slight change in the morphological surface before and after the thermal performance in this experiment. The polymer PLA as a matrix material had a slightly smoother and finer appearance on the continuous and cyclic treatment samples. However, these specimens did not show some deformed shapes (some wrinkle shapes along the longitudinal direction) since it did not undergo sufficiently high-temperature exposure.

**Figure 10:**
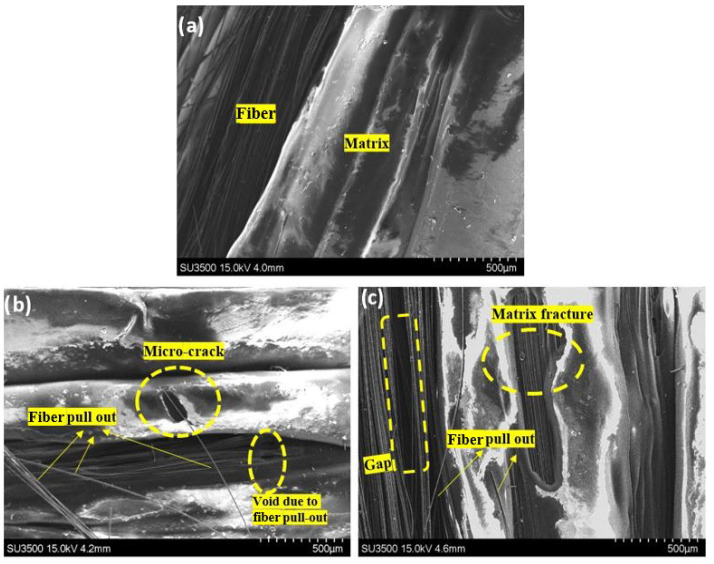
Scanning electron microscope images of the specimen after destructive tensile testing: (**a**) intact (untreated); (**b**) stable continuous at 65 ${}^{\circ }$C; (**c**) cyclic between 50 and 70 ${}^{\circ }$C in a 60${}^{\circ }$, 0${}^{\circ }$, and 90${}^{\circ }$ view angle, respectively.

From the SEM micrographs shown in Figure 10, it can be observed that the effect of thermal cycling and continuous heating on the 3D-printed CFRP specimens caused different microstructural fracture damages after tensile testing in both the polymers and carbon fibers compared to the untreated (intact) specimens. The PLA polymers from the thermally treated specimens were split up with a relatively micro and massive cracked hole located in the middle for continuous and cyclic heating, respectively. As for the intact sample, its polymer structure remained morphologically unseparated, and fracture did not exist. The presented micro-crack in the matrix material was one of the main damage mechanisms caused by the thermal cycles.

## 4. Discussion

Based on the experimental results of the tensile test, the untreated group displayed superior strength results when compared to the heated group subjected to stable continuous and cyclic temperatures. The mechanical response after stable temperature exposure showed a moderate loss in tensile strength and Young’s modulus. The behavior trends of Young’s modulus and the tensile strength were almost similar with a higher detrimental effect under thermal cycling. The mechanical strength of 3D CFRP specimens subjected to the continuous temperature and the cyclic temperature was reduced by 3.6% and 9.6%, respectively. The Young’s modulus of the specimens subjected to the continuous temperature and the cyclic temperature was decreased by 11.4% and 26.5%, respectively. These values obtained from the tensile testing indicated the degradation of the mechanical properties after the thermal exposures.

It was decided to investigate the fracture interface of the 3D-printed samples in order to monitor the deformation behavior and how the fracture happened prior to and after mechanical testing. To study such occurrences, an optical microscope was employed to investigate the interfacial adhesion between the continuous carbon fiber (CCF) and PLA thermoplastic matrix prior to destructive tensile testing. The SEM microscope was used to examine the fracture interface of the 3D CFRP specimens and changes in the matrix microstructure from each group. To investigate such outcomes, one specimen from each group was chosen that best reflected the failure mode.

The thermal cycling performed in this work was with an oxidative condition, where environmental air was used during the cooling process by opening the ventilator to the maximum scale to allow the air to enter the oven chamber. This oxidative atmosphere led to accelerated aging of the CFRP composites due to a mismatch in the thermal expansion coefficient (CTE), which caused the polymer matrix’s oxidation and cracking [18,19]. While microscopic-scale damage such as micro-cracking occurred as a result of the impact and internal stresses, micro-cracking was the leading cause of material failure due to its undetectable nature, as well as the induced structure fragmentation, which led to a reduction in the mechanical properties such as strength, stiffness, and dimensional stability [29].

The visual investigation of the failure area of the 15 specimens was studied in accordance with ASTM standard D3039. The failure mode of each thermal group is presented in Figure 8. It was indicated that the intact or untreated specimen group exhibited the lateral-at tab-top (LAT) failure mode and well-agreed with the result reported by researchers [27]. The intact sample after longitudinal tension loading exhibited lateral fiber breakage and fiber splitting completely at the top region near the tab, which was gripped by the gauge. Edge delamination in the middle (DGM) was the nominated failure mode in the stable continuous heated group’s specimen. It can be seen that there was debonding between layers in the middle due to the lower strength of interfacial adhesion formed after the continuous thermal loading for 6 h at 65 ${}^{\circ }$C. The explosive breakage at gauge in the middle (XGM) failure mode occurred in the sample from the treated group after thermal exposure with cycling mode between 50 ${}^{\circ }$C and 70 ${}^{\circ }$C for six cycles, and this result was similar to what was studied by Ghasemi et al. [28]. In this damage mode, a large amount of fibers pulled out near the gauge, and it was presumed to be due to the reduced chain scission and cross-linking of the polymer matrix caused by the thermal cycling. Moreover, it can be observed from Figure 8c that layer debonding also occurred after the thermal cycling.

The appearance of breakage phenomena can be also triggered by the interfacial forces exerted between the fiber and the matrix, as presented in Figure 8. It has been recently reported in research work where functionalized surfaces with pure cellulose nanocrystals [30] are faced with composites made by matrices with a polar nature and cellulose-rich hemp/flax fibers [31]. These studies concluded that the breakage of bundles and the single hemp/flax fibers was due to these high interfacial forces. In addition, the early stages of cycling were also linked to the production of microvoids, followed by interfacial sliding, which caused composite quality degradation [32].

The mechanical properties of polymeric composites are more matrix-dependent, making them more susceptible to thermal aging. However, it is worth noting that the type of thermal loading can also influence composite behavior. As mentioned previously, the detrimental effect on the mechanical behavior of the 3D CFRP composite was attributed to the difference of the coefficient of thermal expansion (CTE) between the matrix and fiber, and it was caused by the reduced cross-linking of the polymers [25]. This CTE disparity caused local thermal stress, which might cause fiber pull-out due to fiber–matrix debonding. The debonding of fiber and matrix occurs in an oxidative environment due to the significant strain gradients present in matrix locations near highly stiff fibers, causing high local stresses, which lead to crack initiation and mechanical deterioration of composite specimens [18,19,26].

The visual inspection of the morphological surface shown in Figure 9 showed that there was a slight change to the morphological surface before and after the thermal treatment in this experiment. On stable (prolonged) and cyclic treatment samples, the polymer PLA as the matrix material had a slightly smoother and finer appearance. However, because they were not exposed to sufficiently high temperatures, these specimens did not exhibit visible warpage or deformed shapes (some wrinkle shapes along the longitudinal direction). Moreover, the warpage of the 3D CFRP specimen was governed by the stress level released in the macroscopic dimension [33]. It can be observed from Figure 10 that matrix cracks occurred after the thermally stable treatment, and in the cycled group, the crack was larger. When the specimens were exposed to stable heating (prolonged) for 6 h at 65 ${}^{\circ }$C, which is a few degrees above the glass transition temperature of the PLA polymer used, the molecular chains of the polymer changed their structure into an amorphous one, and only local motions of stretching occurred [20]. In addition to that, the prolonged heating allowed the formation of adsorptive inter-layer bonds and volatile groups. As a result, the polymer structure changed, and inner stress relaxation occurred as a result of this thermo-process, which is known as thermo-relaxation [34]. In the case of thermal cycling, the strain gradients were accumulated in the matrix material, and the local stresses increased as a result of the reheating and re-cooling exposure of the polymeric composites; the molecular motion of the long-range polymer structure was reactivated several times.

## 5. Conclusions

In summary, we investigated the effect of stable and cyclic temperature on the mechanical properties of unidirectional 3D CFRP composites. Static tensile testing was performed to obtain the mechanical strength, Young’s modulus, and failure damage of these composites. The results indicated that unidirectional CFRP composites undergo thermal exposures, revealing a degraded mechanical strength and Young’s modulus under stable temperature at 65 ${}^{\circ }$C and cyclic temperature between 50 and 70 ${}^{\circ }$C. The specimens under thermal cyclic possessed lower mechanical performance compared to the untreated group by having a 9.6% reduction in tensile strength and 26.5% in the elastic modulus. Meanwhile, the 3D CFRP composite group under continuous thermal exposure exhibited a lower decrease of the mechanical properties by having a 2.4% reduction in tensile strength and 11.4% in the elastic modulus. The degradation in the mechanical strength and elastic modulus after thermally stable and cyclic loading was attributed to the difference in the coefficient of thermal expansion (CTE) between the matrix and fiber, which was caused by the reduced cross-linking of the polymers.

Based on morphological investigations using an optical microscope, the failure mode of 15 specimens was studied in accordance with ASTM standard D3039. It was indicated that the intact or untreated specimen group exhibited lateral damage at top failure mode (LAT), the thermally stable group underwent edge delamination in the middle (DGM) as the nominated failure mode, and the explosive breakage at gauge in the middle (XGM) failure mode occurred in the sample from the thermal cycling group.

Based on the SEM observations, the delamination, fiber pull-out, and matrix cracking were the dominant damages in the 3D-printed tensile-tested specimens. The molecular chains of the polymer changed their structure into an amorphous one, and only local motions of stretching occurred when the specimens were exposed to stable heating (prolonged). In the case of thermal cycling, the strain gradients were accumulated in the matrix material, and the local stresses increased as a result of the reheating and re-cooling exposure of the polymeric composites; the molecular motion of the long-range polymer structure was reactivated several times. Micro-cracking in the matrix occurred as a result of internal stresses, which led to material failure and a reduction in the mechanical properties. The temperature had a considerable influence on the matrix material, and connecting together the CFRP components reduced the material’s strength. As a result, it is critical to comprehend temperature-induced reactions in composite materials.

The findings presented in this work offer pathways to investigate further the degradation of 3D composites under several temperature conditions to study the integrity of the structure and the damage mechanism. In future work, similar thermal exposure modes (thermally stable and thermal cycle) will be performed at different magnitudes to provide an extensive horizon of thermal effects on the mechanical characteristics of 3D-printed CFRP composites. Furthermore, investigating the effect of different thermal cycling parameters such as heating and cooling rates and dwell time on the materials’ properties would be beneficial for industrial applications. The thermal degradation of the polymer material used in the additive manufacturing of a composite should be considered to be investigated through rheological and thermogravimetric measurements to understand the thermal behavior of the material during the heating conditions.

Ultimately, understanding the thermal influence at different conditions on the morphological structure of FDM printed materials and their mechanical properties can show the first step to inspecting and eliminating them. These thermal effects may incite researchers to develop techniques of thermal treatment of printed specimens that withstand their mechanical characteristics to be potential applications for the operation of space under various thermal conditions.

## Figures and Tables

**Table 1:** Material properties of continuous carbon fiber and polylactic acid.

	Elastic Strength	Elastic Modulus	Strain at Failure	Density
	(MPa)	(GPa)	(%)	(g/cm^3^)
Fiber	3530	230	1.5	1.76
Matrix	46.6	2.636	1.9	1.17

**Table 2:** Printing parameters.

Parameter	Value
Nozzle diameter	1.5 mm
Extrusion multiplier	0.7
Primary layer height	0.5 mm
Interior infill	100%
Infill pattern	rectilinear
Nozzle temperature	220 ${}^{\circ }$C
Bed temperature	90 ${}^{\circ }$C
Printing speed	3 mm/s

**Table 3:** Dimensions of 3D-printed specimens before and after heating treatment.

Test Group	Before Treatment			After Treatment		
	$L_{o}$	$W_{o}$	$T_{o}$	$L_{t}$	$W_{t}$	$T_{t}$
	(mm)	(mm)	(mm)	(mm)	(mm)	(mm)
Untreated	148.32 ± 1.04	12.65 ± 0.21	2.14 ± 0.02		-	
Stable	149.88 ± 0.28	13.19 ± 0.25	2.18 ± 0.05	no variation		
Cyclic	149.97 ± 0.42	12.95 ± 0.12	2.08 ± 0.02	150.37 ± 0.42	12.1 ± 0.21	1.74 ± 0.07

## Data Availability

Not applicable.
